# Erythrocyte phospho-signalling is dynamically altered during infection with *Plasmodium falciparum*

**DOI:** 10.15698/mic2020.10.733

**Published:** 2020-09-16

**Authors:** Jack D. Adderley, Christian Doerig

**Affiliations:** 1 Centre for Chronic Infectious and Inflammation Disease, Biomedical Sciences Cluster, School of Health and Biomedical Sciences, RMIT University, Bundoora VIC 3083, Australia.

**Keywords:** malaria, protein kinase, kinomics, host-directed therapy HDT, host-pathogen interactions, signaling

## Abstract

It is well established that intracellular pathogens mobilise signalling pathways to manipulate gene expression of their host cell to promote their own survival. Surprisingly, there is evidence that specific host signalling molecules are likewise activated in a-nucleated erythrocytes in response to infection with malaria parasites. In this paper (Adderley *et al.*, Nature Communications 2020), we report the system-wide assessment of host erythrocyte signalling during the course of infection with *Plasmodium falciparum*. This was achieved through the use of antibody microarrays containing >800 antibodies directed against human signalling proteins, which enabled us to interrogate the status of host erythrocyte signalling pathways at the ring, trophozoite and schizont stages of parasite development. This not only confirmed the pre-existing fragmentary data on the activation of a host erythrocyte PAK-MEK pathway, but also identified dynamic changes to many additional signalling elements, with trophozoite-infected erythrocytes displaying the largest mobilisation of host cell signalling. This study generated a comprehensive dataset on the modulation of host erythrocyte signalling during infection with *P. falciparum*, and provides the proof of principle that human protein kinases activated by *Plasmodium* infection represent attractive targets for antimalarial intervention.

## ANTIBODY MICROARRAYS – USEFUL TOOLS TO INVESTIGATE HOST CELL SIGNALLING DURING INFECTION

We published previously that infection with *P. falciparum* results in the activation of a signalling pathway in the host erythrocyte, that involves the human enzymes p21-activated kinase (PAK) and MAPK/ERK kinase (MEK). The fact that PAK isoforms serve as signalling nexuses that control multiple pathways stimulated our interest in addressing the host erythrocyte's signalling response in a system-wide manner. We turned to the antibody microarray technology developed by Kinexus (a Vancouver-based kinomics company) and used an array comprising 878 antibodies directed against human signalling proteins. 613 of these antibodies are phosphorylation specific, which provides information on key activation/inhibitory phosphorylation events. The remaining 265 antibodies are pan-specific, recognising the phosphorylated and unphosphorylated forms of host proteins, thus providing information on changes in abundance. We used the array to compare extracts from uninfected and *P. falciparum*-infected erythrocytes at ring, trophozoite and schizont stages of development. This uncovered ~150 dynamic and significant changes in phosphorylation of erythrocyte signalling proteins, with specific sites modulated at distinct times during parasite development.

## ACTIVATION OF HOST c-MET DURING *P. FALCIPARUM* TROPHOZOITE DEVELOPMENT

**Figure 1:**
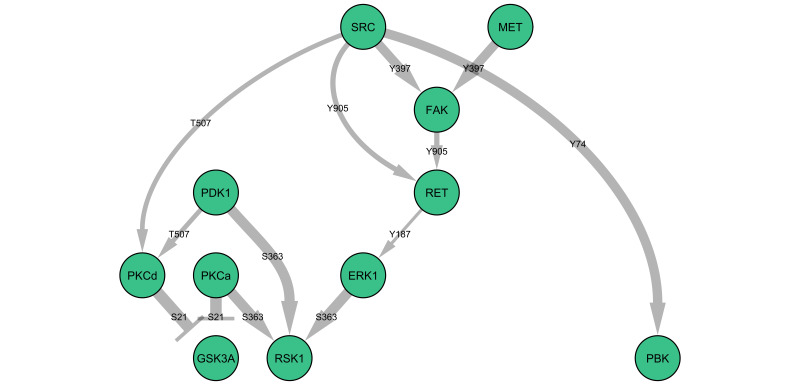
FIGURE 1: Hypothetical connections between host erythrocyte signalling proteins based on antibody microarray data obtained at the trophozoite stage of parasite development. Displayed here are some of the signalling proteins which are interconnected as per information in the literature. Arrow thickness reflects the magnitude of the increase in the signal obtained with a phospho-specific antibody against the indicated amino acid residue.

Of particular interest was striking multi-residue hyper-phosphorylation of the tyrosine kinase c-MET at the trophozoite stage, and of the serine/threonine kinase B-Raf during the entire infection process. We confirmed these results for c-MET and B-Raf using phosphosite-specific antibodies in Western blot analyses. In addition to validating the microarray data for these two proteins, this showed that B-Raf is activated early (at the ring stage) and is subsequently degraded, suggesting that the parasite modulates the activation status of signalling pathway elements not only through phosphorylation, but through controlling the abundance of some of these elements. This is in line with previous reports of the degradation of specific host cell proteins upon infection.

c-MET is a receptor tyrosine kinase, and therefore it is likely to be the initiator of downstream signalling events. **Figure 1** illustrates a potential signalling network downstream of c-MET, derived from the statistically significant changes in phosphorylation of specific proteins (as observed in the microarray experiments) at the trophozoite stage of parasite development. This raises the testable hypothesis that GSK3a and RSK1 activity may be dependent on c-MET activation during infection. Interestingly, c-MET is usually activated through the binding of the extracellular ligand hepatocyte growth factor, which was not present in the cell culture medium used in these experiments; how c-MET is activated during infection is intriguing and remains to be determined. Likewise, elucidating the actual organisation of the pathways triggered by infection (i.e. testing connection hypotheses such as those presented in **Figure 1**) and determining their effector mechanisms now requires targeted work on individual pathways/components.

In addition, we are also undertaking a more holistic approach to understand the host phospho-signalling environment through mathematical modelling, with the aim of further understanding the overall flow of information through the host cell and better interpret the signalling environment.

## INHIBITION OF HOST CELL c-MET AND B-RAF IMPAIRS PARASITE PROLIFERATION

The observation that c-MET and B-Raf (among several other host kinases, see Fig. 2 of the Nature Communications paper) are activated during infection raises the possibility that these enzymes are required for parasite survival. We were indeed able to demonstrate that highly selective inhibitors of c-MET (PHA-665752 and Crizotinib) and B-Raf (SB-590885) display high potency (sub-micromolar) *in vitro* against *P. falciparum* and the phylogenetically distinct human malaria *P. knowlesi*. In addition, we demonstrated that PHA-665752 was also effective against *P. berghei* in the mouse model of malaria. This strongly suggests that these host kinases are essential for *Plasmodium* spp. development within erythrocytes; the multi-species efficiency these molecules display makes them potential leads for further antimalarial development.

## HOST CELL KINASES AS TARGETS FOR HOST-DIRECTED ANTIMALARIAL INTERVENTION

Malaria remains a major global public health problem, with *P. falciparum* being responsible for a vast majority of the ~450,000 annual deaths. Parasites resistant to all deployed antimalarials, including the front-line artemisinin combination therapies, have spread throughout South East Asia and were recently reported in South America, which threatens malaria elimination/eradication efforts. To address this global health challenge, we are in urgent need of next-generation drugs with untapped modes of action (to prevent cross-resistance) and a low propensity to select for *de novo* resistance. In the discussion of the Adderley *et al.* paper, we propose that one potential solution to this problem lies in the innovative field of host-directed therapy (HDT), whose application to parasitic infections still needs to be explored. In HDT, the drug target is an enzyme produced by the host, and which the pathogen requires for survival. The major advantage of HDT over traditional anti-infective drugs that target the pathogen directly is that resistance is slower to emerge, as selection under drug pressure of mutants encoding a mutated, resistant target cannot occur because the latter is not encoded by the pathogen.

We have now demonstrated that a number of host erythrocyte kinases, including (but not restricted to) PAK, MEK, c-MET and B-Raf, are activated by *P. falciparum* infection, and that treatment with specific inhibitors of these human kinases kill the parasite. Human kinase dysregulation plays an important role in oncogenesis, which has stimulated an extensive and still growing kinase-directed cancer drug discovery pipeline. Our findings open novel avenues for antimalarial development, which include the repurposing of clinically approved drugs directed against human targets. This would considerably accelerate the process, and lead to drugs that would have a low propensity for the emergence of resistance.

